# Can breastfeeding protect against antimicrobial resistance?

**DOI:** 10.1186/s12916-020-01862-w

**Published:** 2020-12-15

**Authors:** Maya L. Nadimpalli, Claire D. Bourke, Ruairi C. Robertson, Elisabeth Delarocque-Astagneau, Amee R. Manges, Amy J. Pickering

**Affiliations:** 1 Department of Civil and Environmental Engineering, Tufts University, Science & Engineering Complex, Anderson Hall, Room 204, 200 College Avenue, Medford, MA USA; 2 Stuart B. Levy Center for Integrated Management of Antimicrobial Resistance (Levy CIMAR), Tufts University, Boston, MA USA; 3 Centre for Genomics and Child Health, Blizard Institute, Queen Mary University of London, London, E1 2AT UK; 4 Zvitambo Institute for Maternal and Child Health Research, Harare, Zimbabwe; 5 Université Paris-Saclay, UVSQ, Inserm, CESP, Team Anti-infective Evasion and Pharmacoepidemiology, 78180 Montigny, France; 6 AP-HP, GHU Paris Saclay University, Raymond Poincaré Hospital, Epidemiology and Public Health Department, 92380 Garches, France; 7 School of Population and Public Health, The University of British Columbia, Vancouver, BC Canada; 8 British Columbia Centre for Disease Control, Vancouver, BC Canada

**Keywords:** Breastfeeding, Human milk, Microbiome, Antimicrobial resistance, Child health, Low- and middle-income countries

## Abstract

**Background:**

The proportion of infections among young children that are antimicrobial-resistant is increasing across the globe. Newborns may be colonized with enteric antimicrobial-resistant pathogens early in life, which is a risk factor for infection-related morbidity and mortality. Breastfeeding is actively promoted worldwide for its beneficial impacts on newborn health and gut health. However, the role of breastfeeding and human milk components in mitigating young children’s carriage of antimicrobial-resistant pathogens and antibiotic resistance genes has not been comprehensively explored.

**Main body:**

Here, we review how the act of breastfeeding, early breastfeeding, and/or human milk components, such as the milk microbiota, secretory IgA, human milk oligosaccharides, antimicrobial peptides, and microRNA -bearing extracellular vesicles, could play a role in preventing the establishment of antimicrobial-resistant pathogens in young children’s developing gut microbiomes. We describe findings from recent human studies that support this concept.

**Conclusion:**

Given the projected rise in global morbidity and mortality that will stem from antimicrobial-resistant infections, identifying behavioral or nutritional interventions that could decrease children’s susceptibility to colonization with antimicrobial-resistant pathogens may be one strategy for protecting their health. We suggest that breastfeeding and human milk supplements deserve greater attention as potential preventive measures in the global effort to combat antimicrobial resistance, particularly in low- and middle-income settings.

## Background

Antimicrobial-resistant (AMR) bacterial infections are becoming increasingly common across the globe [1]. As many as 162,000 US adults currently die from multidrug-resistant bacterial infections each year, making resistant infections the third leading cause of death [2]. A 2019 UN report described antimicrobial resistance as a global crisis, with potential for knock-on financial shocks due to increasing healthcare costs, poverty, and inequality comparable to those experienced during the 2008–2009 global financial crisis [3]. Morbidity and mortality stemming from antimicrobial resistance are expected to be highest in low- and middle-income countries (LMICs), where bacterial diseases like lower respiratory infections, diarrheal disease, and tuberculosis remain common.

Infants are particularly susceptible to the potential negative consequences of AMR infections [4]. Evidence suggests that young children may be colonized or infected with enteric AMR pathogens early in life [5–8]. Presence and elevated abundance of AMR pathogens in the intestinal tract are risk factors for transmission to non-enteric sites (e.g., blood, surgical sites) [9–12], horizontal transfer to other individuals, and vertical transmission from mothers to their infants. Neonatal infections with highly resistant pathogens like extended-spectrum β-lactamase (ESBL)-producing *Enterobacteriaceae*, which are considered an urgent health threat by the US Centers for Disease Control and Prevention [13], have been documented across the globe and have become increasingly prevalent over the past decade [14, 15]. Children under two are also one of the likeliest age groups to receive antibiotics [16, 17]. Frequent antibiotic exposures may increase the relative abundance of antibiotic resistance genes (ARGs) harbored by gut bacteria, and foster a gut environment supportive of their transfer to pathogens [4, 18–20].

Breastfeeding has long been recognized for its beneficial impacts on newborn health, including provision of maternal immune cells [21] and antibodies to protect against infection [22] and decreased risk of exposure to diarrheal pathogens from non-breastmilk food-sources [23]. However, to date, few studies have examined how early breastfeeding practices and breastmilk constituents could specifically mitigate young children’s colonization with AMR bacteria and ARGs. In general, breastfed children experience fewer diarrhea-related hospitalizations [23, 24] and may consume fewer antibiotics [24], which helps to prevent exposure to, colonization with, and selection for AMR pathogens and their ARGs [18]. In addition, early and exclusive breastfeeding is increasingly recognized as an integral step in the development of healthy infant microbiomes. While the developing infant gut microbiome is shaped by transmission from multiple maternal body sites [25], human milk induces intestinal tolerance to the commensal microbiome through the simultaneous provision of nutrition for the infant and the bacteria themselves [26]. Furthermore, human milk contains maternal immune effectors (e.g., antibodies, immune cells, and antimicrobial peptides) that enhance pathogen exclusion and elimination, and food and microbial antigens that promote infant immune development. Healthy microbiomes provide benefits into adulthood [27] and may also be better able to resist colonization with pathogens, including AMR pathogens, a phenomenon known as “colonization resistance” [28]. However, the potential links between specific breastfeeding practices or human milk components and children’s reduced susceptibility to colonization or infection with AMR bacteria have not been comprehensively explored.

Here, we argue that breastfeeding may be an important strategy for combatting the global antimicrobial resistance crisis. We begin by reviewing how early breastfeeding, exclusivity of breastfeeding, and components of human milk could provide protection against the establishment, proliferation, and/or selection of enteric pathogens in general and AMR bacteria and ARGs, in particular. We describe the findings of recent observational studies and intervention trials that have explored such associations. Finally, we comment on how escalating antimicrobial resistance and changing breastfeeding trends in LMICs make these settings particularly important for exploring breastfeeding as a way to mitigate antimicrobial resistance. Given the scale and scope of the impending antimicrobial resistance crisis, identifying scalable, cost-effective behavioral or nutritional interventions that could decrease children’s susceptibility to colonization by enteric AMR pathogens and ARGs will be critical to protecting their health.

## Potential benefits of early breastfeeding

The first form of human milk produced by the mammary glands, called “colostrum,” could be especially important in preventing the establishment and proliferation of antibiotic-resistant bacteria in very young children’s intestines. Colostrum contains concentrations of maternal immune cells and secretory IgA (sIgA) that are up to 6× and 12× higher than subsequent human milk, respectively [22, 29]. Maternal leukocytes provide active immunity against gut pathogens by engulfing and killing them, and may provide protection beyond the gut by crossing the intestinal epithelium and entering the bloodstream [21]. IgA antibodies are secreted by lymphocytes in the mammary gland. These lymphocytes have migrated from the mother’s gut, meaning they target antigens and enteric microorganisms to which mothers have previously been exposed. This includes antibodies to pathogens that can be antibiotic-resistant, such as *Shigella* and *Campylobacter* [22, 30]. Once in the gut, sIgA promotes the clearance of targeted pathogens by blocking pathogen access to intestinal epithelial receptors, binding to bacterial adhesion sites like pili, trapping them in mucus, opsonizing them for detection by Fc-receptor-bearing immune cells, and promoting their removal [31, 32]. In addition, sIgA can directly neutralize bacterial virulence factors and toxins, including *Escherichia coli* heat-labile enterotoxin [31, 33]. Higher concentrations of sIgA antibodies in colostrum and human milk are associated with greater protection against pathogen-mediated diarrhea [33, 34], and could clearly provide early protection against children’s colonization or infection with AMR bacteria. Additionally, early breastfeeding increases the likelihood of exclusive breastfeeding [35], which could confer distinct protection against young children’s colonization with AMR bacteria and ARGs, as outlined below.

## Potential benefits of exclusive or predominant breastfeeding for first 6 months

**Fig. 1:**
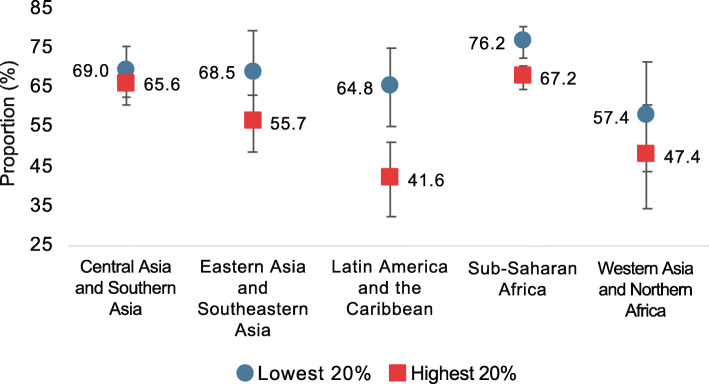
Average proportion of children in low- and lower middle-income countries (LMICs) predominately receiving human milk at 0–5 months, by wealth quintile and region, 2010–2017. In LMICs in Latin America, the Caribbean, and Sub-Saharan Africa in particular, babies from the wealthiest families are substantially less likely to be predominately breastfed than babies from the poorest families. Analysis is based on a subset of 66 countries with recent (2010–2017) data for predominant breastfeeding at 0–5 months. Country income classifications provided in source dataset. Error bars show the 90% confidence interval around the mean. *Source*: United Nations Children’s Fund, Division of Data Research and Policy. *Global UNICEF Global Databases: Infant and Young Child Feeding: Predominant breastfeeding* (2019)

**Fig. 2:**
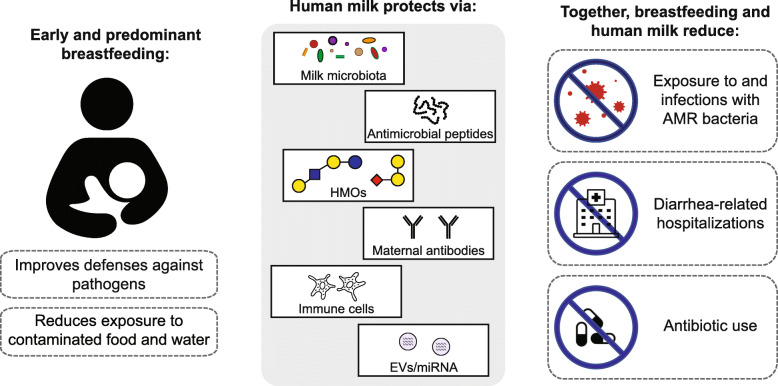
Ways breastfeeding and human milk could prevent the establishment, proliferation, and/or selection of antimicrobial-resistant bacteria among young children. Exclusive or predominant breastfeeding for an extended duration improves children’s defenses against pathogens and reduces their food- and waterborne exposures to antimicrobial-resistant bacteria. Human milk contains multiple components that could protect against antimicrobial-resistant pathogens and reduce the lateral transfer of antibiotic resistance genes. The concentrations of some of these components are highest in colostrum, the earliest form of human milk. HMOs, human milk oligosaccharides; EVs, extracellular vesicles; miRNA, microRNA; AMR, antimicrobial resistance

The World Health Organization (WHO) recommends exclusive breastfeeding through the first 6 months of life. While this is not practiced by the majority of mothers in high-income countries (~ 25% of infants in the USA and Europe are exclusively breastfed through 6 months) [36, 37], rates of exclusive and predominant breastfeeding are higher and increasing in countries with the lowest income status [38], although substantial variability exists (Fig. 1). Exclusive or predominant breastfeeding for an extended duration could indirectly protect children against colonization with AMR pathogens and ARGs in several ways **(**Fig. 2**)**.

### Reduced exposure to antibiotics

Breastfeeding may reduce antibiotic use among children. Recent trials in Finland [39] and France [24] have identified strong inverse relationships between the duration of breastfeeding and frequency of children’s antibiotic use. In France, any breastfeeding at all in the first 6 months was associated with less frequent antibiotic use over the first 2 years of life compared with those who were never breastfed, and longer duration of exclusive or predominant breastfeeding was associated with even lower risks [24]. However, the authors could not exclude that lower antibiotic use may have been due to differences in health-seeking behaviors between breastfeeding versus non-breastfeeding parents. In settings where early-life enteric pathogen exposure is common, breastfeeding may reduce antibiotic use by reducing children’s risk of diarrhea [40]. In LMICs, children’s diarrhea is frequently treated with antibiotics. A review of children’s antibiotic use over the first 2 years of life in eight LMICs found that children were given a median of 3.92 antibiotic courses per child-year, with especially high rates among children in South Asia [19]. By the age of 5, children are estimated to have received an average of 24.5 antibiotic prescriptions in total for treating infections, a rate 5 times higher than observed in high-income countries [20]. At a population level, chronic antibiotic use accelerates the acquisition of ARGs among pathogenic bacteria [41]. Among individuals, antibiotic use can increase gut susceptibility to colonization with virulent pathogens, especially in the hospital setting [42]. By protecting children against diarrhea and improving overall health, breastfeeding may reduce total antibiotic use, reduce diarrhea-related hospitalizations, and prevent associated harms to children’s gut microbiomes during this critical window of development [4].

### Reduced exposure to water- and food-borne AMR bacteria

Children who receive their hydration and nutrition via human milk have reduced early-life exposures to water and foods that may be contaminated with AMR bacteria. Potential bacterial contamination of drinking water and feeding bottles is one of many reasons why formula feeding is discouraged by the WHO. A recent study found that formula feeding may in fact increase the relative abundance of ARG-carrying pathogens, including *Staphylococcus aureus*, *Staphyloccoccus epidermidis*, *Klebsiella pneumoniae*, *Klebsiella oxytoca*, and *Clostridioides difficile* in the gut of preterm infants, compared to preterm infants receiving human milk or fortified human milk [43]. Once children begin eating complementary foods, they can also be exposed to AMR fecal bacteria that contaminate vegetables and meat, especially in LMIC settings [44, 45]. Preventing children’s food- and waterborne exposures to AMR bacteria is critically important during their first few months of life, particularly in regions where the burden of enteric pathogen exposure is high. Impaired gut microbiome development in these settings may compromise the development of colonization resistance against the establishment and proliferation of pathogens [26, 40]. Exclusive or predominant breastfeeding for an extended duration likely confers the greatest protection, though any level of breastfeeding could theoretically reduce children’s environmental exposures to water- and food-borne AMR bacteria.

### Protection by human milk

Human milk constituents can directly protect against the acquisition, establishment, and proliferation of AMR pathogens and ARGs in children’s guts by seeding the gut with non-pathogenic commensals that competitively exclude pathogens and reduce their negative impacts on the host [28, 46–50]; targeting and killing AMR pathogens [21, 48, 51–53]; suppressing gut inflammation which is conducive to pathogen colonization and the lateral transfer of ARGs to unrelated, neighboring bacteria (i.e., via horizontal gene transfer) [47, 54–56]; improving intestinal barrier function to prevent pathogen establishment and translocation [22, 50, 57, 58]; and priming the infant immune system to develop robust responses against exogenous bacteria, including AMR pathogens [50, 59, 60]. Some key protective components beyond sIgA (described above) are reviewed below.

#### Milk microbiota

Human milk contains approximately 10^6^ bacteria/mL and is a continuous source of commensal, mutualistic, and/or potentially probiotic bacteria to the developing infant’s gut [50]. Dominant genera include *Staphylococcus* and *Streptococcus* with hundreds of other taxa identified through 16S rRNA sequencing [61]. Human milk microbiota communities vary between women and over time, and likely comprise both maternal-origin bacteria and infant oral cavity bacteria that enter the milk duct via suckling [62]. Human milk and infant gut microbiomes share a number of commensal organisms by the first week of life [63, 64], with breastfeeding duration and exclusivity influencing these associations in a dose-dependent manner [65]. However, human milk-derived species become less dominant in infants’ guts by the first month [64], suggesting that these initial colonizers could have a specific, early role in infant immune development [25, 66]. Human milk bacteria may protect newborns from AMR pathogen colonization and infection through competitive exclusion of exogenous pathogens, production of antimicrobial compounds, and by enhancing intestinal cell mucin production, which reduces intestinal permeability and improves intestinal barrier function [50, 67]. For example, strains of *Lactobacillus rhamnosus* isolated from human milk have been demonstrated to be effective in inhibiting a wide range of pathogenic bacteria in vivo [68], including multidrug-resistant *E. coli* [69]. Finally, it is hypothesized that human milk bacteria prime the infant immune system by training intestinal cells to respond appropriately to commensals versus harmful pathogens [50]. Development of a robust immune response is key to preventing AMR pathogen proliferation and infection in the developing child.

A substantial portion of the human milk microbiota is thought to comprise non-pathogenic maternal gut bacteria that have been endogenously transported from the mother’s intestinal lumen to the mammary glands during late pregnancy and lactation [61]. Because maternal-origin bacteria are particularly stable in the infant gut [25], it has been proposed that modulating the maternal gut microbiome during pregnancy and/or lactation could positively impact the development of the infant gut microbiome [61]. To our knowledge, associations between maternal gut microbiome modifications and increased infant resilience against AMR pathogen colonization or infection have not been directly explored.

#### Human milk oligosaccharides (HMOs)

Human milk contains high levels of HMOs, a family of structurally diverse unconjugated glycans that are uniquely found in human milk and thought to shape the composition of the developing infant gut microbiome. HMO levels vary between mothers and over time, depending on the stage of lactation [70]. A high concentration of HMOs in the early infant gut could reduce children’s susceptibility to gut colonization by AMR pathogens and ARGs through multiple mechanisms.

First, HMOs are an ideal substrate for *Bifidobacterium* species, which can dominate the infant gut and may prevent the establishment and proliferation of AMR bacteria and enteric pathogens [27, 46, 48]. *Bifidobacterium* species like *Bifidobacterium longum* subsp*. infantis* efficiently consume HMOs [47], creating acidic by-products which reduce intestinal pH. This lower pH environment is unfavorable for the growth of *Enterobacteriaceae*, which commonly encode ARGs [46]. Multiple *Enterobacteriaceae* genera, including all ESBL-producing *Enterobacteriaceae* and drug-resistant *Salmonella* and *Shigella*, are considered critical antibiotic resistance threats by the US Centers for Disease Control and Prevention [71]. *Bifidobacteria*, on the other hand, much less commonly encode transferable ARGs [27, 72]. *Bifidobacteria* also produce bacteriocins, which exhibit antimicrobial activity against enteric pathogens and bacteria frequently resistant to antibiotics, such as *Escherichia coli* [48, 53]. In addition to suppressing their growth, specific *Bifidobacterium* species have been demonstrated to prevent the lateral exchange of ARGs between neighboring *Enterobacteriaceae* in mouse models [73]. Previous research among a cohort of Bangladeshi children has demonstrated inverse, dose-response relationships between infants’ gut levels of *Bifidobacteria* and concentrations of both ARGs and *Enterobacteriaceae* species [27].

Second, HMOs may directly neutralize invading AMR pathogens and reduce gut inflammation, which otherwise causes a favorable environment for *Enterobacteriaceae* growth and ARG transfer [55]. The structure of HMOs is similar to cell surface glycoconjugates used by enteric pathogens to bind and enter target cells. Thus, HMOs can prevent the adhesion of enteric pathogens to the intestinal mucosa by serving as alternate binding sites [51]. Further, there is some evidence that HMOs can directly inhibit the expression of genes involved in inflammation, although the mechanisms remain unclear [56]. *B. infantis*, which thrives in gut environments with high HMO levels, has also been shown to suppress the production of proinflammatory cytokines and is associated with lower concentrations of fecal calprotectin—a marker of intestinal inflammation—throughout the first 2 months of life [47, 54] The capacity for HMOs and breastfed gut environments to suppress gut inflammation is critical in preventing growth blooms of harmful AMR pathogens, including AMR *Enterobacteriaceae* [74].

#### Antimicrobial factors

In addition to sIgA and HMOs described above (see the “Potential benefits of early breastfeeding” section and previous section, respectively), human milk contains many other factors that directly inhibit AMR pathogens and/or their adhesion to epithelial cell surfaces. These include lactoferrin, an iron-binding protein with antimicrobial properties that is present in high concentrations in human milk, especially colostrum. Lactoferrin can be extremely effective in preventing the proliferation of *E. coli* [52], and has also been demonstrated to prevent the growth, impair the virulence, and prevent the epithelial attachment of many other bacterial pathogens via multiple modes of action [75], including *Shigella* and *Salmonella* [51, 57]. Recent randomized control trials among infants in LMICs have suggested that lactoferrin supplementation of children’s feeds could reduce risks of neonatal sepsis [76] and reduce the duration of diarrheal illness [77]. In addition, other proteins present in human milk, once they bind to free-floating oligosaccharides, can prevent the adhesion of enteric pathogens and/or their toxins to the intestinal lumen via similar mechanisms to sIgA, HMOs, and lactoferrin. The glycosylated human milk proteins MUC1 and MUC4 are effective against frequently AMR enteric pathogens like *Salmonella*, for example [51]. β-casein, a common glycosylated human milk protein, has also been shown in mouse models to also stimulate the expression of MUC2 genes and increase the number of mucus-producing goblet cells and Paneth cells in the small intestine. The development of a healthy intestinal mucus layer is critical in preventing the establishment of AMR pathogens, protecting the gut epithelium against pathogen-mediated damage, and promoting pathogen clearance [75]. Lactoferrin and other antimicrobial factors in human milk are present at the highest concentrations directly post-partum [78].

Other antimicrobial proteins may enhance the effectiveness of antibiotics against AMR pathogens. For example, the human milk factor HAMLET (human α-lactalbumin made lethal to tumor cells) has been shown to directly target as well as enhance the effectiveness of antibiotics against drug-resistant *Staphylococcus aureus* and pathogenic streptococci [79, 80], which could reduce the duration of antibiotic therapy needed to treat an infection and thereby minimize associated harms to infants’ developing microbiomes [4]. Recent experimental studies suggest that other antimicrobial peptides present in human milk, such as β-defensins, may also be able to work in synergy with antibiotics to lower the minimum inhibitory concentrations of intracellular pathogens [81].

There is great interest in developing human milk glycoconjugate supplements that could help support or reinforce natural mechanisms of protection against neonatal infection [51]. Whether these compounds could also protect children against colonization or infection with AMR bacteria merits further investigation.

#### Extracellular vesicles (EVs)

The potential role of human milk EVs, particularly exosomes, in regulating infants’ gut maturation has only recently gained attention [82, 83]. EVs are non-replicating cell membrane-bound microvesicles (20–2000 nm) naturally produced by almost every mammalian cell. They are believed to be critical in cell-to-cell signaling as they can bear surface receptors or ligands and encapsulate and transport cell contents. EVs secreted into human milk are capable of surviving harsh conditions, including digestion, and appear to be a stable transport mechanism for the transfer of maternal-origin microRNA (miRNA) to infant intestinal cells [83]. miRNAs are small (22 nucleotide length) non-coding RNAs that control gene expression, largely by binding to and accelerating the degradation of messenger RNA. A handful of miRNAs may account for the majority of miRNAs delivered by human milk EVs [83], and recent transcriptomic studies indicate these miRNAs may help protect gut epithelial cells from enteric infection while the immune system is still maturing [58]. Cell surface receptors harbored by human milk EVs may also modulate the developing infant immune system and direct EVs to certain cell types [84]; recent studies have demonstrated that these EVs promote intestinal epithelial cell growth and appear to protect intestinal epithelial cells from oxidative stress [85, 86]. Although human milk EV research is still in its infancy, there is already great interest in determining whether EV supplementation could help protect newborns against colonization or infection with enteric pathogens [82], including AMR pathogens.

## Evidence from human studies for the protective benefits of breastfeeding against infants’ colonization by AMR pathogens

Several randomized controlled trials, and observational and mouse model studies have explored the effectiveness of human milk components (e.g., sIgA [30, 31, 33, 34], lactoferrin [51, 52, 57], other glycoconjugates [51]) and human milk-supported taxa (e.g., *Bifidobacteria* [48, 53]) against enteric pathogens in general. However, to date, fewer studies have examined the potential protective effects of breastfeeding and human milk supplements against AMR bacteria and ARGs specifically [27, 54, 87–91].

Several observational studies have identified a potential protective effect of current breastfeeding against healthy children’s acquisition and/or colonization with enteric AMR bacteria. A longitudinal study of 100 healthy newborns in Spain found that current breastfeeding was protective against first acquisition of ESBL-producing *Enterobacteriaceae* (adjusted hazard ratio (aHR), 0.29; 95% confidence interval (CI), 0.11, 0.80) [89]. Consumption of any human milk and exclusive breastfeeding were also found to be protective against enteric colonization with ESBL-producing *Enterobacteriaceae* in relatively small cross-sectional studies conducted in rural Venezuela (*n* = 78) [90] and Lebanon (*n* = 117) [91], respectively. Other epidemiological studies have identified a protective effect of exclusive or predominant breastfeeding on children’s infections with enteric pathogens that are commonly drug-resistant, including nontyphoidal *Salmonella* [45, 92] and *Campylobacter* [30, 93].

Whether breastfeeding is protective against AMR pathogen colonization among already hospitalized children remains unclear. A cross-sectional study of infants admitted to a hospital in rural Kenya found that current breastfeeding was not protective against gut colonization with ESBL-producing *E. coli* at the time of admission nor during hospitalization [94]. A 5-year study of infants hospitalized in a neonatal intensive care unit in Italy also found that breastfeeding was not protective against ESBL-producing *E. coli* acquisition [95]. However, breastfed infants were less likely to become colonized with multidrug-resistant gram-negative bacteria during their stay. Further, among children who did acquire multidrug-resistant gram-negative bacteria, more frequent breastfeeding appeared to be protective against their colonization by multiple species (versus only one) [95].

Recent metagenomic sequencing studies have described associations between breastfeeding and children’s developing gut microbiomes and resistomes, defined as all ARGs present across bacterial species. In Europe, a mother-infant paired study by Pärnänen et al. found that early termination of exclusive breastfeeding was associated with higher relative gut abundance of multiple ARGs and the bacterial taxa that typically encode them (e.g., *Gammaproteobacteria*) in infants’ guts, compared to infants who were exclusively breastfed through 6 months of age [87]. Specifically, the authors reported that early termination of all breastfeeding (i.e., prior to 6 months) was associated with the increased relative abundance of ARGs conferring resistance to aminoglycoside, sulfonamide, and tetracycline in infants’ resistomes, as well as higher relative abundance of mobile genetic elements that could allow these ARGs to be transferred to pathogens via horizontal gene transfer. Findings from a separate sequencing study of infants’ stool suggest that HMO-supported *Bifidobacteria* growth could mediate these associations [27]. The authors found that a gut microbiome predominantly made up of *Bifidobacteria* spp. was associated with significant reductions in both the number and relative abundance of ARGs present in children’s guts by 1 year of age, compared to infant microbiomes with low abundance of *Bifidobacteria* spp. The authors observed that *Bifidobacteria* dominance likely suppressed the growth of common ARG-encoding taxa (an observation that has been demonstrated in mouse models) [46]. As described above, HMOs in human milk provide an ideal substrate for *Bifidobacterium* species.

A recent randomized control trial among healthy newborns in the USA has provided the most robust evidence to date of the potential for human milk supplements to protect infants against AMR pathogen colonization [88]. The authors demonstrated that daily supplementation during the first month of life with a *B. infantis* strain that relies on HMOs for colonization significantly reduced the relative abundance of ARGs (by 90%) and the bacterial genera commonly associated with them (i.e., *Escherichia*, *Staphylococcus*, *Bacteroides*, and *Clostridioides*), strongly indicating that HMO-supported growth of *Bifidobacteria* could be critical to promoting breastfed infants’ gut resilience to AMR pathogens and ARGs [88]. Because HMO diversity and concentrations in human milk can vary between mothers and over time [70], daily *Bifidobacteria* supplementation could be a way to ensure high *Bifidobacteria* abundance in the guts of breastfed children, and possibly a way to extend health benefits among children who are only partially breastfed during the first months of life. To our knowledge, no randomized trials have explored whether improved breastfeeding practices or early-life supplementation of other human milk components could directly protect young children from colonization with ARGs or AMR pathogens of critical concern.

## Human milk as a source of antimicrobial resistance

Although breastfeeding likely cultivates a gut environment that is ultimately protective against children’s colonization and infection with AMR pathogens, human milk may also be source of AMR bacteria and ARGs to the developing infant [87, 96]. In a recent study of 16 mother-infant pairs by Pärnänen et al., approximately 70% of ARGs and 76% of bacterial species detected in mothers’ human milk were also detected in their infants’ guts, suggesting vertical transmission [87]. The same study observed that maternal antibiotic prophylaxis during delivery was associated with both a higher number and abundance of ARGs in breastfed infants’ guts for up to 6 months, indicating that maternal antibiotic exposures may have lasting impacts on breastfed infants’ gut resistomes [87]. AMR *Staphylococcus*, *Streptococcus*, *Acinetobacter*, *Enterococcus*, and *Corynebacterium* strains have also been cultured from human milk [96]. AMR bacteria and ARGs detected in human milk likely do not originate solely from the mammary glands, as bacteria from mothers’ skin and infants’ mouths (through retrograde inoculation during suckling) are also thought to contribute to the milk microbiota [50]. Nevertheless, human milk appears to be one of many sources of AMR bacteria and ARGs to the developing infant. This could be consequential if antibiotic use during delivery becomes more common to prevent maternal and neonatal infections, particularly in LMIC settings [97, 98].

## Exploring breastfeeding as a strategy to combat antimicrobial resistance

The potential benefits outlined here suggest that breastfeeding should be actively explored as a scalable, cost-effective strategy to address antimicrobial resistance, in addition to the many other health benefits that breastfeeding confers [23]. In high-income countries, where less than half of newborns receive any human milk at 6 months [23], improving optimal breastfeeding practices could reduce early-life antibiotic use and improve gut colonization resistance against AMR pathogens, which are becoming more common in the community. If breastfeeding or human milk constituents are shown to be protective against colonization or infection with AMR bacteria, this would be one of many important reasons to accelerate efforts to promote breastfeeding at the policy level.

Breastfeeding may be an especially important strategy to explore in LMICs. Due to a higher prevalence of bacterial disease, LMICs are expected to be disproportionately burdened by the antimicrobial resistance crisis [99]. Although absolute antibiotic use has historically been higher in high-income settings due to healthcare access [100], antibiotic consumption is increasing in LMICs and appropriate targeting of antibiotics is limited by widespread informal antibiotic use and restricted resources for identifying, treating, and preventing AMR infections [99, 101]. By 2050, up to 90% of AMR-related mortality is predicted to occur in countries in Asia and Africa [1], most of which are low- or middle-income. Although systematic investigations are sparse, several studies of healthy children in LMICs [5, 7, 102] report colonization with enteric multidrug-resistant gram-negative bacteria at rates that far exceed what is observed in the USA and Europe. For example, 82% of 1–2-year-old children in Bangladesh [103] were recently reported to be colonized with third-generation cephalosporin-resistant *Enterobacteriaceae*, compared to 4.5% of similarly aged children in the USA [104] and ~ 14% in Switzerland [105]. Household members’ hands, household soil, drinking water, flies, and food have all previously been implicated as sources of both human- and animal-origin fecal bacteria [44, 45, 106–109].

Globally, breastfeeding rates continue to be the highest in countries with the lowest income status [23]. However, within LMICs, differences exist based on mothers’ location (urban versus rural), education status, and household income. If these differences persist, future breastfeeding rates could be impacted. For example, multiple studies have demonstrated that mothers in urban settings exclusively breastfeed their children for a shorter duration of time than rural mothers [110–112]. A recent study from India, a rapidly developing LMIC, suggests that this difference is most pronounced among urban mothers who have at least a primary school education (compared to urban mothers with no formal education), suggesting that the increased employment opportunities afforded by education, and/or greater access to formula and the money to purchase it, may contribute to these differences [110]. Further, fewer wealthy mothers living in LMIC exclusively or predominantly breastfeed their children during their first 6 months of life compared to mothers belonging to the lowest wealth quintiles (Fig. 1) [113]. These discrepancies widen as children grow; 20–23-month-old babies from the poorest families continue to receive at least some breast milk at rates that are 1.3–2.8 times higher than those from the wealthiest families [114]. Collectively, these trends suggest that breastfeeding practices could worsen in LMICs with strong breastfeeding traditions as they urbanize and transition to greater per capita wealth [23, 110]. This may be a particularly important public health concern in settings where reductions in environmental pathogen exposure lag behind wealth- and education-associated reductions in breastfeeding, as a higher fraction of pathogens will be AMR in the coming decades. Policies aimed at promoting breastfeeding in LMICs should find ways to support mothers, such that the benefits for infant health are maximized while mothers’ access to new educational and economic opportunities afforded by economic transition is maintained.

## Conclusion

Multiple strategies have been proposed to prevent the coming global antimicrobial resistance emergency, including the development of new antibiotics and vaccines, improved surveillance and education, and improved hospital infection control [1]. Given that AMR pathogens are increasingly prevalent in community settings, early-life interventions like breastfeeding that could protect children against community-acquired gut colonization and infection should also be actively explored. Recent evidence supports the role of breastfeeding in preventing the acquisition, establishment, and proliferation of enteric pathogens, including AMR bacteria, in young children’s guts by reducing children’s early-life exposures to contaminated foods and inappropriate antibiotic use. The emerging mechanistic pathways through which human milk components including the milk microbiota, maternal antibodies, HMOs, antimicrobial peptides, and EVs may protect against early-life colonization by AMR pathogens and ARGs warrant further exploration as novel targets for intervention, and/or supplementation where optimal breastfeeding practices are not possible. The evidence presented here suggests that breastfeeding and human milk supplements deserve greater attention as potential preventive measures in the global effort to combat AMR.

## Data Availability

The datasets analyzed to generate Fig. 1 of the current study are available from the United Nations Children’s Fund, Division of Data Research and Policy: https://data.unicef.org/resources/dataset/infant-young-child-feeding/.

## Abbreviations

AMR: Antimicrobial-resistant
ARG: Antibiotic resistance gene
HMO: Human milk oligosaccharide
LMIC: Low- and middle-income country
EV: Extracellular vesicle
miRNA: MicroRNA
sIgA: Secretory IgA
WHO: World Health Organization
